# Assessment of the oral health behavior, knowledge and status among dental and medical undergraduate students: a cross-sectional study

**DOI:** 10.1186/s12903-019-0716-6

**Published:** 2019-01-29

**Authors:** Ke Yao, Yufei Yao, Xin Shen, Changqing Lu, Qiang Guo

**Affiliations:** 1 State Key Laboratory of Oral Diseases, National Clinical Research Center for Oral Diseases, West China Hospital of Stomatology, Sichuan University, Chengdu, China; 2 Department of Anatomy, West China School of Basic Medical and Forensic Medicine, Sichuan University, Chengdu, China

**Keywords:** Oral health knowledge, Oral health behavior, Oral health status, Oral health education, Dental students, Medical students, China

## Abstract

**Background:**

It is widely accepted that oral health plays an important role in overall health. Both dental and medical students are expected to possess good oral health awareness and work together for public oral health promotion especially in developing countries like China. The aim of this study was to assess the oral health knowledge, behavior and status of dental and medical undergraduate students in the first (fresh) and third year (before specialized courses) study.

**Methods:**

A self-administered structured questionnaire with 13 questions was designed based on oral health knowledge, behavior and status and a cross-sectional study was conducted among the 1st, 3rd year dental students (1DS, 3DS) and medical students (1MS, 3MS) of Sichuan University in Chengdu, China, in the period of September–December 2017. The data was analyzed by chi-square test using IBM SPSS Statistics v. 21.0.

**Results:**

The oral health behavior, consciousness and status of the 1st, 3rd year medical and dental students were not optimistic. Dental freshmen were slightly superior to the medical ones in terms of the brushing methods and the awareness of oral disease-systemic disease relationship. The junior dental students showed highly significant improvement than their counterparts, mainly in the items about frequency of brushing teeth, brushing methods of vertical scrub or Bass technique (66.3%), usage of floss or mouth wash (49.7%), causes of caries, periodontal diseases and system diseases (56.9–83.4%). The rates mentioned above were 36.1, 15.8%, 26.7–43.6% among 3MS, respectively. In terms of oral health status, significant differences were only observed in junior students. The prevalence rates of bad breath, gum bleeding, and tooth discoloration among 3DS were obviously lower than those of 3MS. However, only a total of 17.2% junior students had a good oral health, including 23.8% dental students and 11.4% medical students.

**Conclusions:**

Our study provided a new understanding of oral health knowledge, behavior and status among dental and medical students, which may help to promote the reform of oral health education and establish a model for clinicians and dentists to work together for improving oral health.

## Background

Healthy mouth is a unique and priceless treasure, and it is regarded as a fundamental human right to maintain a good oral health [1]. Oral health is traditionally defined as an oral status that is free of diseases, which not only makes people look beautiful, but also contributes to the normal function of mouth [2]. In 2016, the Federal Dental International (FDI) Dental World Federation redefined the oral health comprehensively, recognizing that oral health was multifaceted and involved the ability to smell, touch, taste, chew, swallow, smile, speak, and convey a lot of emotions through facial expressions with confidence and without discomfort, pain, and disease of the craniofacial region [3].

Oral health plays an important role in overall health and is an indispensable part of general health [4–6]. It is reported that there is a close relationship between oral diseases and other systemic diseases like diabetes, digestive disease, stroke, cardiovascular disease, metabolic syndrome, adverse pregnancy outcomes, obesity, et al [7–10]. On the one hand, oral problems could result in a pro-inflammatory state, where systemic diseases might develop [11, 12]. On the other hand, systemic disorders might be responsible for the development of oral problems [13, 14]. However, oral health care is always neglected in despite of the importance of oral health in general health [15, 16]. Oral diseases are still one kind of the most prevalent problems that affect the overall health of human being [17]. Periodontitis and dental caries, as two major oral problems, affects 60 and 36% people worldwide, respectively [18–20]. Surprisingly, according to the recently issued data acquired from the 4th national oral health epidemiology survey of China, the caries prevalence rates of children aged 5 and 12 were 70.1 and 34.5%, respectively, even higher than those reported by the 3rd national survey in 2005 [21].

Given the importance of oral health in the whole body and the high prevalence of oral diseases, the joint effort of dentists and clinicians is essential to people’s health, and it should be integrated as a part of the comprehensive health promotion [22, 23]. In addition, the cognition and behavior of oral health professionals reflect their understanding of oral preventive measures and practices, which have a great impact on their delivery of oral health care and then affect the oral health of patients [24, 25]. Therefore, it is very important for dental and medical students to have a good oral health awareness as they will be the major providers of health services and be responsible for public oral health promotion in the future. The improved awareness of oral health amongst dental students is beneficial to the maintenance of patients’ oral health and is instrumental in preventing oral diseases [26]. In comparison with dental students, medical students sometimes are more likely to encounter underserved and vulnerable patients [27]. As the providers of primary health care for the majority of patients, medical professionals are also expected to play a part in oral health promotion [28]. Thereby, medical students are expected to master optimal oral health knowledge and awareness so as to provide patients with necessary oral health instruction when needed.

People’s oral health knowledge, behavior and status are influenced by many factors including culture, environment and social customs, et al [29–31]. There were some reports about oral health knowledge and behavior of dental students or medical students in other countries [2, 24, 32, 33], but little was known in China. Hence, the present study was conducted to assess the oral knowledge, behavior and status of dental and medical students in Chengdu, China, in order to provide basic data for the oral health education for undergraduates, especially dental and medical students.

## Methods

### Recruitment of study subjects

The first and third-year dental students and medical students from West China School of Stomatology and West China School of Medicine of Sichuan University, respectively, were invited to take part in the study using a self-administered structured questionnaire. The ethics committee of West China School of Stomatology, Sichuan University approved this study. All the students understood the nature and purpose of the survey and were told how to fill in the questionnaire. The students completed the questionnaires under supervision, and the questionnaires were collected immediately after completion.

### Questionnaire design

**Fig. 1:**
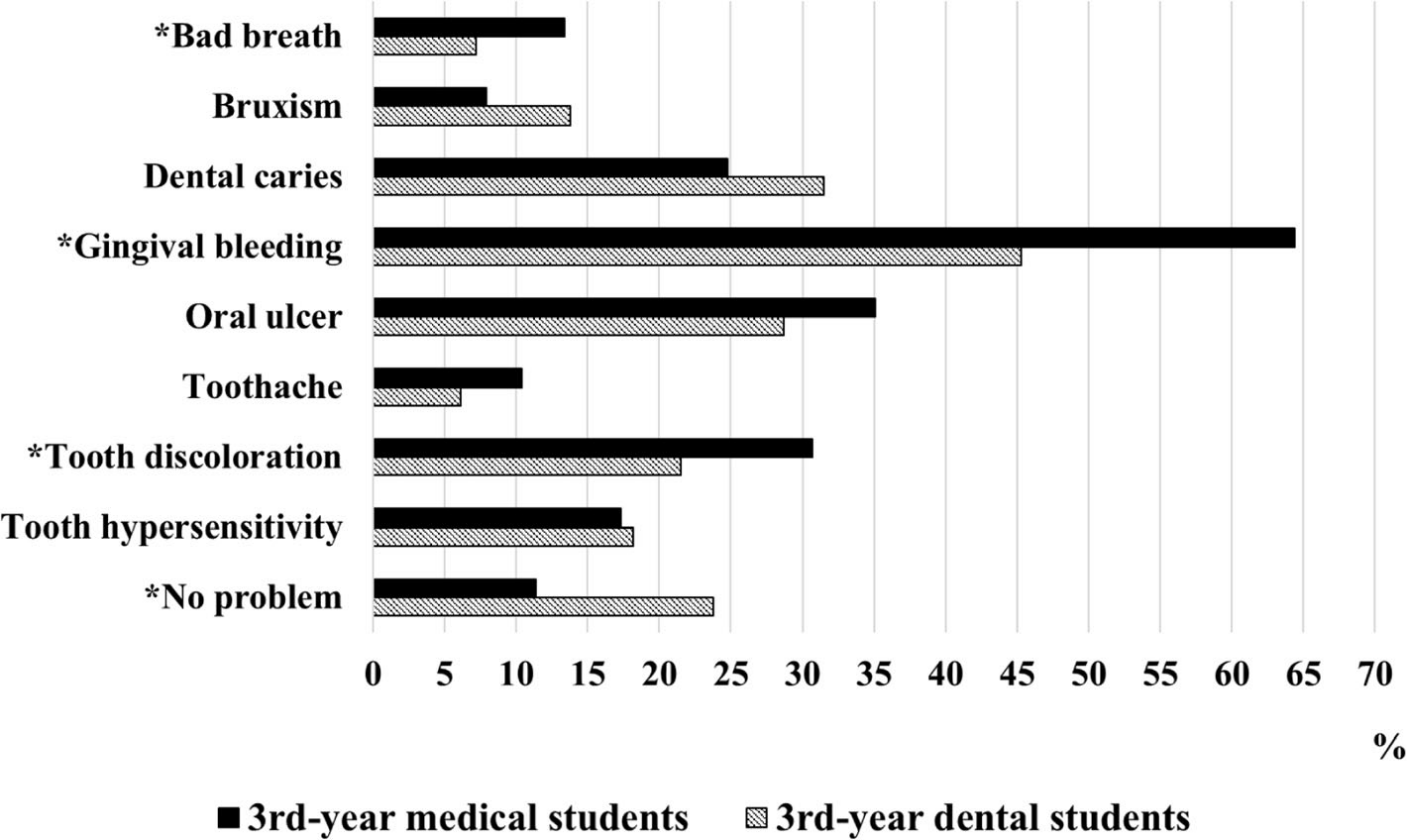
Oral health status of dental and medical students in the 3rd year (%). Pearson’s Chi-square test was used. *P value was less than 0.05

The demographic information of participants including specialty, grade, and gender and ethnicity was requested to be filled at first. The questionnaire consisted of 13 questions, which were designed to evaluate oral health behavior, knowledge and status of dental students and medical students in the first and third year and was divided into three parts. The first part was oral health behavior survey, including 6 questions (options for question 5 and 6 are shown in Table 2) 1) how many times do you brush your teeth every day (once or less, twice, three times or more), 2) how long do you brush your teeth every time (1 min or less, 2 min, 3 min or more), 3) how often do you replace your toothbrush (3 months or less, about half a year, never until it cannot be used), 4) when do you visit the dentist (regularly, once suffer from oral diseases, after an oral disease lasts for a long time, until life quality is greatly impacted by oral diseases), 5) how do you brush your teeth, and 6) which oral hygiene methods besides tooth brushing below do you use in your daily life. In the second part, oral health knowledge was studied by 6 questions (options for each question are shown in Table 3) 1) which are the causes of dental caries, 2) which are the causes of bleeding during tooth brushing, 3) which is the influence of dental plaque, 4) which measures can prevent oral diseases, 5) which systemic diseases may be related to oral diseases, and 6) which is more important for keeping good oral health: self-administration or dentists. Finally, we investigated the oral health status by asking students what oral problems they had (Fig. 1). Students were allowed to choose more than one options in several questions.

### Statistical analysis

The collected data was analyzed by IBM SPSS Statistics v. 21.0 (IBM, Armonk, NY, USA). Pearson’s Chi-square test was used to compare the proportions, and Fisher’s exact test was adopted if necessary. A *P*-value less than 0.05 was considered to be statistically significant.

## Results

### Subjects

In total, 850 questionnaires were distributed and 774 copies were collected. Demographic characteristics of the subjects were shown in Table 1. Gender, age and ethnicity were matched and had no significant difference (data not shown).

**Table 1:** Demographic characteristics of subjects participated in the survey (n)

Grade	Dental students	Medical students	Total
1st year	190	201	391
3rd year	181	202	383
Total	371	403	774

### Oral health behavior

As shown in Table 2, there were few significant differences in freshmen’s oral health behavior, except for the better habits of dental students in duration of tooth brushing and the method of tooth brushing. In contrast, the junior dental students were much better than their counterparts at almost every behavior. More third-year dental students brushed their teeth twice or more every day, for no less than 2 minutes every time, replaced toothbrush every 3 months and visited the dentist regularly. As for the method of tooth brushing, 66.3% of the third-year dental students followed the right procedure of the vertical scrub or modified Bass technique, whereas more than half of the third-year medical students wrongly brushed their teeth horizontally or irregularly. On the other hand, 49.7% (40.9, 23.8%, respectively) of junior dental students cleaned their mouth by dental floss or mouth wash, which were much higher than the ratio of 15.8% (6.4, 11.4%, respectively) in junior medical students. To our surprise, 80.1% of the third-year dental students put off visiting the dentist until they were afflicted by oral diseases, as only 19.9 of them visited the dentist regularly.

**Table 2:** Oral health behavior of dental and medical students in 1st and 3rd year (%)^a^

Question (recommended behavior)	1st year			3rd year		
	Dental students	Medical students	P	Dental students	Medical students	P
Frequency of daily tooth brushing (≥ twice)	93.2	89.6	0.206	97.8	87.1	**0.000**
Duration of tooth brushing (≥ 2 min)	94.7	83.1	**0.000**	96.7	84.2	**0.000**
Frequency of replacing toothbrush (≤ 3 months)	66.8	69.7	0.551	71.3	59.4	**0.015**
Frequency of visiting the dentist (regularly)	10.5	5.5	0.065	19.9	10.4	**0.009**
Method of tooth brushing						
Vertical scrub	38.4	24.4	**0.003**	35.4	29.7	0.238
Horizontal scrub	11.1	24.9	**0.000**	6.1	22.3	**0.000**
Modified Bass technique	20.5	6.5	**0.000**	30.9	6.4	**0.000**
Fones technique	10.5	3.5	**0.006**	9.9	6.4	0.209
Irregular	19.5	40.8	**0.000**	17.7	35.1	**0.000**
Oral hygiene methods besides tooth brushing						
Dental floss	14.2	3.5	**0.000**	40.9	6.4	**0.000**
Mouthwash	13.7	14.9	0.726	23.8	11.4	**0.001**
Sugar-free chewing gum	11.1	11.4	0.903	4.4	5.0	0.806
Toothpick	7.4	6.5	0.726	4.4	8.4	0.114
None	64.7	65.7	0.846	47.0	74.3	**0.000**

^a^Pearson’s Chi-square test, bold numbers meant *P* < 0.05

### Oral health knowledge

As shown in Table 3, dental students had a better knowledge in the causes of caries and periodontal diseases than medical students especially in grade 3. For example, more than half dental students thought that dysbiosis of oral microflora (58.6%) and inadequate tooth brushing (52.5%) could cause dental caries, and that fluoride application (66.9%) and pit and fissure sealing (84.5%) could prevent caries, while in medical students, these ratios were less than 50%. There was a similar trend in the understanding of periodontal disease. 79.0 and 79.6% of the third-year dental students thought that bleeding while brushing teeth or dental plaque correlated with periodontal disease, however, these ratios in medical students were only 47.0 and 61.9%, respectively. Unfortunately, no enough attention was paid by medical students, whether they are freshmen or juniors, to the issue that systemic diseases (such as heart disease, diabetes mellitus, hypertension, cancer, etc.) might be related to oral diseases.

**Table 3:** Oral health knowledge of dental and medical students in 1st and 3rd year (%)^a^

Question	1st year			3rd year		
	Dental students	Medical students	P	Dental students	Medical students	P
Causes of dental caries						
Toothpaste without fluoride	10.0	16.4	0.062	15.5	13.9	0.657
Frequent ingestion of sugar	52.6	55.2	0.607	60.2	55.9	0.397
Dysbiosis of oral microflora	30.5	26.9	0.424	58.6	41.1	**0.001**
Inadequate tooth brushing	41.6	41.3	0.954	52.5	40.6	**0.020**
Don’t know	3.7	1.0	0.151	2.8	3.0	0.903
Causes of bleeding during tooth brushing						
Natural physiological phenomenon	7.4	6.5	0.726	3.3	8.9	**0.024**
Periodontal disease	35.3	36.3	0.828	79.0	47.0	**0.000**
Brushing too hard	37.4	35.3	0.674	33.7	38.6	0.318
Excessive internal heat^b^	38.9	53.7	**0.003**	36.5	37.1	0.893
Systemic disease	4.2	2.5	0.342	23.8	20.3	0.414
Don’t know	3.2	2.0	0.681	1.1	3.0	0.359
Influence of dental plaque						
Affecting appearance	36.3	40.8	0.363	45.9	37.1	0.083
Inducing dental caries	22.6	38.8	**0.001**	59.7	35.6	**0.000**
Inducing periodontal disease	67.4	51.2	**0.001**	79.6	61.9	**0.000**
No big deal	3.7	2.0	0.311	1.1	9.9	**0.000**
Don’t know	6.8	5.5	0.573	1.7	3.0	0.611
Measures that prevent oral diseases						
Application of fluoride	50.5	13.9	**0.000**	66.9	23.3	**0.000**
Pit and fissure sealing	72.6	16.9	**0.000**	84.5	25.7	**0.000**
Tooth scaling	86.3	90.5	0.190	97.8	91.6	**0.008**
Don’t know	2.6	3.0	0.833	1.7	2.0	1.000
Systemic diseases that may be related to oral diseases						
Heart disease	31.6	10.9	**0.000**	56.9	29.2	**0.000**
Diabetes mellitus	34.7	13.9	**0.009**	83.4	43.6	**0.000**
Hypertension	21.6	11.4	**0.007**	57.5	26.7	**0.000**
Cancer	32.1	19.4	**0.004**	60.8	34.7	**0.000**
None of the above	8.9	19.9	**0.002**	3.3	10.9	**0.004**
Other diseases	21.1	47.3	**0.000**	30.4	23.8	0.144
Don’t know	11.6	2.0	**0.000**	0.6	2.0	0.437
Which is more important for oral health: self-administration or dentist?						
Self-administration of oral hygiene	48.9	49.3	0.952	40.3	55.9	**0.002**
Regular visit to dentist	51.1	50.7		59.7	44.1	

^a^Pearson’s Chi-square test, bold numbers meant *P* < 0.05

^b^Excessive internal heat: a concept in traditional Chinese medicine (TCM). People with excessive internal heat would have an increased risk of inflammation, sore throat, oral ulcer, acne, etc

### Oral health status

With regard to oral health status, there was no significant difference between the first-year dental students and medical students (data not shown). The rate of self-reported oral health was about 14%. On the other hand, the third-year dental students had lower prevalence rate than their medical counterparts in some aspects, such as bad breath, gingival bleeding, and tooth discoloration (Fig. 1). To our surprise, the proportions of the third-year dental students (3DS) and the third-year medical students (3MS) with periodontal disease were 45.3 and 64.4%, respectively, if using self-reported gum bleeding as a diagnostic criterion. What’s more, less than a fifth of students in total thought they had a good oral health (data not shown). According to the self-reported questionnaire, dental students had a healthier mouth, accounting for 23.8%, compared with 11.4% for medical students.

## Discussion

Since dental and medical students are the health professionals of the future, they are expected to possess accurate oral health knowledge and behavior in their school years. At the same time, their oral health status, which not only affects their own health and life quality, but also is a reflection of their oral health attitude and behavior, is remarkably important. Therefore, it is essential to find out their oral health knowledge, behavior and status, which are of great significance to themselves and patients.

The first-year dental and medical students involved in our study haven’t received any oral health-related education and training. Although the professional courses are set in the 4th year, the 3rd-year dental students in our study have received more than 2 years of oral disease-related basic research training/experiment courses and oral health instruction. By collection and comparison of the self-reported questionnaires, we aimed to unveil the oral health-related situation in dental and medical schools in China and provide a reference for education reform.

In oral health behavior, even in the freshmen, approximately 90% of students brushed their teeth at least twice a day, which was much higher than the ratio of 36.1% of middle-aged people revealed in the fourth national survey of China [21] and even higher than those of dental students in other four Asian countries [33], indicating that, to some extent, the oral health behavior of China’s new generation was improving compared to their elders. We also found that dental participants performed better than the medical in oral health knowledge, such as questions with regard to the influence of plaque, measures that prevent oral diseases and systemic diseases that may be related to oral diseases, and the result was similar to previous studies, which suggested that dental students had more knowledge about oral health than others including medical students [2, 29]. This may be explained by the fact that dental students had gained more oral health education from various ways in dental college even though they hadn’t had professional courses yet in the third year, which equipped them with more oral health knowledge than their peers. Moreover, they are more willing to search for relevant knowledge on their own initiative, as majority of them will take on the job as a dentist in the future. However, it was interesting to know that, as a whole, the first-year dental students seemed to know more about oral health than medical freshmen, despite the fact that they were both newly enrolled in university and had received few lectures on oral health in school. The difference may originate from their varied backgrounds and interests. For instance, some dental students are born in dentist families and exposed to more oral health education. In addition, the ambition to become a dentist may have inspired them to learn more knowledge of oral health since their childhood. Though oral health knowledge of dental students was better than that of medical students, both of them did not reach the desirable level. Even among juniors, about 40% students used wrong methods to brush their teeth. There were more than 40% of junior students who weren’t aware of the critical role that bacterium and plaque played in the pathogenesis of caries, and about four fifths of them didn’t realize that gum bleeding may be a symptom of other systemic diseases. The rate of regular oral examination was only 20%, which was lower than those disclosed in previous studies [34, 35]. Considering their pivotal role in health education, therefore, it is necessary to take some measures to enhance their oral health knowledge.

In oral health knowledge, more dental students (over 70%) had a good perception of the issues of “plaque inducing periodontal disease” and “bleeding gums suggesting periodontal disease” than medical students (around 50%) in grade 3. Actually, 90% of bleeding gums are caused by periodontitis or gingivitis, and a few of them are caused by systemic diseases. With the improvement of people’s living standard, the ratio of gum bleeding caused by lack of vitamin or excessive internal heat has decreased dramatically. Poor cognition in the etiology of periodontal disease resulted in a high prevalence of gum bleeding in 3MS (64.4%). Although it was much lower than the rate of middle-aged people (87.4%) [21], periodontal problems should not be underestimated. Periodontal disease is a risk factor for cardiovascular disease, diabetes, cancer, hypertension and so on, which posed a great threat to human health [36, 37]. Freshmen, as well as junior medical students, had a significant lack of understanding of this problem. It seems periodontal disease and systemic diseases are associated with each other in-depth. Oral examination and scaling, in addition to adequate tooth brushing and regular use of dental floss, are effective methods to prevent periodontal disease. So it is more important to keep a positive health awareness and behavior in daily life.

It was noteworthy in our study that only 14.6% of freshmen and 23.8% of 3DS, respectively, claimed to be free of oral problems. For example, 56.8 and 40.3% of dental students in grade 1 and 3, respectively, admitted that their gums tended to bleed when they brushed their teeth, which were much higher than those in other study [24]. Although dental students were superior to their medical companions in many aspects, both of them needed to strengthen their knowledge, attitude, behavior of oral health and finally to be competent to improve the oral health status of their patients and themselves. It was reported that clinicians were overwhelmingly willing to participate in oral health care, but it was hindered by the fact that they had never been instructed formally in oral-health-related subjects before and were short of the knowledge and skills needed [22, 38–40]. The role clinicians could play in improving the oral hygiene practices of the patients could be enhanced by reinforcing oral health component of the medical curriculums [32]. This would empower the clinicians and reflect on their participation in oral health education activities. Ultimately, it could potentially result in improved oral health knowledge, preventive practices and oral cavity condition, especially in rural areas. For dental students, these prevention-related courses could be set as early as possible, furnishing them with enough knowledge and making them conscious of the significance of oral health at the very beginning of professional lives. On the other hand, educators should take the responsibility to elevate students’ knowledge and awareness of oral health including preventive measures, and to advance the translation of knowledge into stable behavior regardless of their personal characteristics [41–43].

The dental school and medical school of Sichuan University rank, respectively, as one of several best schools in China and the students involved in our study come from all around China, where the oral health philosophies may not be the same in different regions. And 774 copies out of 850 questionnaires were recovered, producing a response rate as 91.1% in all. The findings of the present study, thus, are reliable and may reflect the situation of oral health knowledge, behavior, and status of Chinese dental and medical students. Nevertheless, the results should be interpreted with some limitations in mind. Although anonymous questionnaires were adopted, the self-reported design might introduce bias on account of the socially desirable answers. Furthermore, its cross-sectional design was unable to clarify the causation of differences. Future study should be committed to unveiling the potential causality. Analyses of factors that conduce to the changes could boost the development of strategies which would improve students’ oral health knowledge, behavior and status.

## Conclusions

Our study revealed that although dental students performed better than medical ones, both of them need to improve their knowledge, behavior and status of oral health. More emphasis should be placed on methods of tooth brushing, oral cleaning measures, awareness of periodontal disease-systemic disease relationship, regular oral examination, and then oral health maintenance.

## Abbreviations

1DS: The first-year dental students
1MS: The first-year medical students
3DS: The third-year dental students
3MS: The third-year medical students
FDI: Federal Dental International
TCM: Traditional Chinese medicine
